# Disruption of Germination and Seedling Development in *Brassica napus* by Mutations Causing Severe Seed Hormonal Imbalance

**DOI:** 10.3389/fpls.2016.00322

**Published:** 2016-03-15

**Authors:** Tung C. T. Nguyen, Christian Obermeier, Wolfgang Friedt, Suzanne R. Abrams, Rod J. Snowdon

**Affiliations:** ^1^Department of Plant Breeding, IFZ Research Centre for BioSystems, Land Use and Nutrition, Justus Liebig UniversityGiessen, Germany; ^2^Department of Chemistry, University of SaskatchewanSaskatoon, SK, Canada

**Keywords:** *Brassica napus*, oilseed rape, seeds, hormones, germination, vigor

## Abstract

The *Brassica napus* (oilseed rape) accession 1012-98 shows a disturbed germination phenotype that was thought to be associated with its lack of testa pigmentation and thin seed coat. Here, we demonstrate that the disturbed germination and seedling development are actually due to independent mutations that disrupt the balance of hormone metabolites and their regulators in the seeds. High-throughput UPLC-MS/MS hormone profiling of seeds and seedlings before and after germination revealed that 1012-98 has a severely disturbed hormone balance with extremely atypical, excessive quantities of auxin and ABA metabolites. The resulting hypersensitivity to abscisic acid (ABA) and a corresponding increase in dormancy often results in death of the embryo after imbibition or high frequencies of disturbed, often lethal developmental phenotypes, resembling *Arabidopsis* mutants for the auxin regulatory factor gene *ARF10* or the auxin-overproducing transgenic line iaaM-OX. Molecular cloning of *Brassica ARF10* orthologs revealed four loci in normal *B. napus*, two derived from the *Brassica* A genome and two from the C genome. On the other hand, the phenotypic mutant 1012-98 exhibited amplification of C-genome *BnaC.ARF10* copy number along with a chimeric allele originating from recombination between homeologous A and C genome loci which lead to minor increase of *Bna.ARF10* transcription on the critical timepoint for seed germination, the indirect regulator of *ABI3*, the germinative inhibitor. *Bna.GH3.5* expression was upregulated to conjugate free auxin to IAA-asp between 2 and 6 DAS. Functional amino acid changes were also found in important DNA binding domains of one *BnaC.ARF10* locus, suggesting that regulatory changes in *Bna.ARF10* are collectively responsible for the observed phenotpyes in 1012-98. To our knowledge, this study is the first to report disruption of germination and seedling development in *Brassica napus* caused by the crosstalk of auxin-ABA and the corresponding regulators *Bna.ARF10* and *Bna.GH3.5*.

## Introduction

Seed germination and seedling establishment are crucial processes in life cycles of seed plants. In fact, strong seedling vigor or rapid seedling growth is a major breeding target in *Brassica oleracea* (Bettey et al., 2000), rice, and other crops (Redoña and Mackill, 1995) as seedling vigor is closely associated with crop growth and yield (Ellis, 1992). Unfortunately, these traits are polygenic (Bettey et al., 2000) and determined by the interaction of hormonal (Holdsworth et al., 2008), genetic, and environmental components (Bentsink and Koornneef, 2009). Several plant hormones, including abscisic acid (ABA), gibberellins (GA), ethylene, brassinosteroids (BR), auxin and cytokinins (CK), interact to regulate germination (Kucera et al., 2005). The two prominent hormones in dormancy and germination control are the germinative inhibitor ABA and the germination promoter GA, respectively. Generally, ABA biosynthesis and sensitivity increases during seed development and maturation to prevent premature germination, whereas, GA accumulation and sensitivity dominate after seed imbibition, promoting the transition to germination (Hilhorst and Karssen, 1992; Finkelstein et al., 2008). Crosstalk at molecular levels of hormonal signaling influences the ABA:GA balance and thereby promotes or inhibits germination (Kucera et al., 2005; Finch-Savage and Leubner-Metzger, 2006; Finkelstein et al., 2008). Besides the ABA:GA balance, the individual sensitivity of the seed to these two hormones also plays a role in regulating dormancy and germination status (Finkelstein et al., 2008). Germination is preceded by a decrease in ABA levels resulting from the activation of ABA hydroxylation predominantly at C-8′ position to 8-hydroxy ABA, PA, and DPA and of conjugation to ABA glucosylester (Nambara and Marion-Poll, 2005). In fact, endogenous ABA contents decreased significantly within 6–24 h after the onset of imbibition in *Arabidopsis* (Ali-Rachedi et al., 2004) and rice seeds (Ye et al., 2011). In non-endospermic *Brassica* seeds, ABA does not inhibit testa rupture, but inhibits subsequent radicle growth of germinating process (Schopfer and Plachy, 1984; Kucera et al., 2005). Germinating seeds of lettuce (*Latuca sativa* L.) transiently accumulate high levels of ABA-GE and an increase of GA_1_ to support germination and post-germinative growth (Chiwocha et al., 2003). Recent studies have suggested the potential involvement of auxin in regulation of seed dormancy and germination. For example, exogenous application of auxin enhanced the inhibition of seed germination by ABA in *Arabidopsis* via *ABSCISIC ACID INSENSITIVE 3* (*ABI3*) transcription factor (Brady et al., 2003; Liu et al., 2007, 2013) and also delayed seed germination of wheat (Ramaih et al., 2003). When auxin signaling is inactivated by low auxin level or signaling disruption, *Auxin Response Factor 10* (*ARF10*) and *Auxin Response Factor 16* (*ARF16*) are inactivated by the Aux/IAA repressors AXR2 and AXR3. *ABI3* expression cannot be maintained, and seed dormancy is released. With high auxin content or signaling activation, auxin binds to the auxin receptor TIR1/AFB F-box proteins and promotes the degradation of IAA7/AXR2 and IAA17/AXR3. The degradation releases the activity of *ARF10* and *ARF16* and maintains the expression of *ABI3*, which protects seed dormancy and inhibits seed germination (Liu et al., 2013). Under the effect of ABA on auxin-responsive genes, not only *ARF* genes but also early auxin-inducible *GH3.5* and *GH3.6* (for *GRETCHENHAGEN-3*) genes repress the *Arabidopsis* embryonic axis after germination by enhancing auxin signaling (Belin et al., 2009). Stronger dormancy and germination failure were observed in both auxin-overproducing transgenic *Arabidopsis* line *iaaM-OX* (Cheng et al., 2006) and exogenous auxin-applied wild-type *Arabidopsis* seeds (Liu et al., 2007, 2013). In addition, the auxin overproduction mutants *iaaM* and *YUC1-OX Arabidopsis* express long hypocotyls and epinastic cotyledons (Zhao, 2010).

Oilseed rape (*Brassica napus* L., 2*n* = 38, genome AC), a recent amphidiploid species derived from interspecific hybridizations between *Brassica rapa* L. (2*n* = 20, genome A) and *B. oleracea* L. (2*n* = 18, genome C) is the second most important oilseed crop worldwide. Germination and seedling vigor are highly important aspects of seed quality, with a major impact on stand establishment and subsequent yield. Considering the economic importance of germination and vigor, these traits are still poorly understood in oilseed rape and many other important crops. Transfer of knowledge from related model systems into important crops like oilseed rape will greatly increase our ability to improve and manipulate germination and vigor through breeding.

The *B. napus* line 1012-98 is of particular interest for oilseed rape breeding because it has a thin seed coat resulting in reduced dietary fiber content (Badani et al., 2006). This considerably improves the feed and protein quality of rapeseed meal after oil extraction.However, 1012-98 also exhibits negative agronomic characters including a reduced germination rate and inhibition of seedling development and vigor. In contrast to 1012-98, the homozygous inbred line Express 617 has a normal seed coat, germinates normally and shows normal seedling development. To investigate the relationship between seed quality traits, germination and seedling vigor, we generated homozygous, microspore-derived doubled-haploid (DH) progenies from the cross between 1012-98 and Express 617 and screened these for seedling vigor traits and metabolic profiling. Regulation of germination and seed-to-seedling transition by phytohormones was investigated in the parental lines and their DH progenies by high-throughput UPLC-MS/MS profiling of hormone metabolites before and after germination. Molecular cloning and expression analysis of *B. napus* orthologs of the auxin-responsive genes *ARF10, GH3.5*, and *GH3.6* were performed to identify functional sequence polymorphisms with a potential impact on seed hormone regulation.

## Materials and methods

### Plant materials

The *B. napus* homozygous inbred line Express 617 was derived by repeated self-pollination of the German winter oilseed rape variety Express (NPZ Lembke, Germany). Express 617 shows normal seed quality, germination, and vigor. The *B. napus* line 1012-98 is a progeny of an interspecific hybrid derived by embryo rescue-assisted resynthesize from an interspecific cross between *B. rapa* and *B. oleracea*. Due to the high relatedness of the *Brassica* A and C genomes, the chromosomes of resynthesized *B. napus* frequently contain non-reciprocal homeologous translocations (Udall et al., 2005) that can lead to replacement or recombination of homeologous gene copies in either the A or C genome. 1012-98 shows a yellow-seeded phenotype, atypical for *B. napus*. This phenotype is primarily attributable to a major quantitative trait locus (QTL) on chromosome A09 that influences testa thickness and flavonoid pigmentation (Snowdon et al., 2010) and is thought to be caused by a non-homologous translocation leading to gene loss-of-function.

A population of 166 homozygous doubled haploid (DH) lines was generated by microspore culture (Weber et al., 2005) from a single F1 plant derived from the cross between Express 617 and 1012-98. This segregating population was used to investigate the inheritance of the germination and vigor phenotypes of 1012-98. All seeds used for the investigations were harvested from self-pollinated plants grown under normal field conditions in a common environment. For determination of correlations between germination and seed quality traits, contents of fiber components and the seed color were screened by near-infrared spectrophotometry using calibrations developed by Wittkop et al. (2009).

### Germination and seedling development

Germination rate was assessed *in vitro* according to the recommendations of the International Seed Testing Association (ISTA, 2010). In each of three replications a total of 100 seeds each from Express 617, 1012-98, and the 166 DH lines were imbibed on moistened filter paper in Jacobsen germination vessels filled with 50 ml distilled water. Seeds were germinated in a growth chamber at a constant temperature of 25°C with 55% relative humidity and a photoperiod of 16 h light/8 h darkness.

Seedling development was assessed in three repetitions of 27 soil-sown seeds per genotype grown under controlled conditions in a climate-controlled greenhouse. Total seedling (root and shoot) biomass and hypocotyl length were measured at 7 and 14 days after sowing (DAS). Mean trait values were calculated from all successfully germinated seeds per genotype.

### Sampling for hormone analysis and quantitative RT-PCR

For comparison of hormone metabolite profiles and differential expressions of *B. napus Bna.ARF10, Bna.GH3.5*, and *Bna.GH3.6* in Express 617 and 1012-98, 10 identical Jacobsen germination pots per genotype were prepared for sampling every 24 h from 1 to 8 days after sowing (DAS) and every 48 h from 8 to 12 DAS. The experiment was conducted in three replications, total of 15 (ca. 50 mg), and 50 (ca. 100 mg) seeds/seedlings per genotype and replication were pooled into 15 ml Falcon tubes, immediately immersed in liquid nitrogen and lyophilized for 24 h for hormone analysis and quantitative RT-PCR, respectively.

### Internal standards for hormone quantification

Calibration curves and quality controls for dihydrophaseic acid (DPA), abscisic acid glucose ester (ABA-GE), phaseic acid (PA), 7′-hydroxy-ABA (7′-OH-ABA), neo-phaseic acid (neoPA), and indole-3-acetic acid glutamate (IAA-glu) were created by the Plant Biotechnology Institute of the National Research Council of Canada (PBI-NRC, Saskatoon, SK, Canada). Details on all internal standards used for quantification of hormone metabolites are provided in Supplementary Table S1.

### Extraction and purification of hormone metabolites

Lyophilized plant tissue was homogenized in a bead mill for 2–6 min. A 100 μl aliquot containing all the internal standards, each at a concentration of 0.2 pg μ^−1^, was added to around 50 mg of homogenized tissue. After addition of 3 ml of isopropanol:water:glacial acetic acid (80:19:1, v/v) the samples were agitated for 24 h at 4°C. Samples were then centrifuged and the supernatant was isolated and dried on a Buechi Syncore Polyvap (Buechi, Switzerland). Samples were reconstituted in 100 μl acidified methanol, adjusted to 1 ml with acidified water, and then partitioned against 2 ml hexane. After 30 min, the hexane layer was removed and the hexane partitioning was repeated. The aqueous layer was then isolated and dried. Dry samples were reconstituted in 800 μl acidified methanol and adjusted to 1 ml with acidified water. The reconstituted samples were passed through equilibrated Sep-Pak C18 cartridges (Waters, Mississauga, ON, Canada), the eluate being dried on a centrifugal evaporator. An internal standard blank was prepared with 100 μl mixture of the deuterated internal standards. A QC standard was prepared by adding 20 ng of each analyte to 100 μl of the internal standard. Finally, all samples, blanks and QCs were reconstituted in a solution of 40% methanol (v/v), containing 0.5% acetic acid, and 100 pg μl of each of the recovery standards.

### Hormone quantification by HPLC-ESI-MS/MS

The procedure for quantification of multiple hormones and metabolites, including auxins (IAA, IAA-asp, and IAA-glu), abscisic acid, and metabolites (ABA, PA, DPA, 7′-OH-ABA, neoPA, and ABA-GE), and CKs (2iP, iPA, Z, ZR, dhZ, dhZR, and Z-O-Glu) has been described in detail by Chiwocha et al. (2003; 2005). Samples were injected onto a Genesis C18 HPLC column (100 · 2.1 mm, 4 lm, Chromatographic Specialties, Brockville, ON, Canada) and separated by a gradient elution of water against an increasing percentage of acetonitrile that contained 0.04% acetic acid. Calibration curves were generated from the MRM signals obtained from standard solutions based on the ratio of the chromatographic peak area for each analyte to that of the corresponding internal standard, as described by Ross et al. (2004). QC samples, internal standard blanks, and solvent blanks were prepared and analyzed along with each batch of tissue samples. Mean minimum limits of quantification (LOQ) for each analyte were: 8 ng g^−1^ dry weight (DW) for Z, dhZ, Z-O-Glu, 2iP, iPA, and ABA; 60 ng g^−1^ DW for ZR, dhZR, IAA-asp, IAA-glu, IAA, and 7′-OH-ABA; 118 ng g^−1^ DWfor DPA; 78 ng g^−1^ DW for PA; 56 ng g^−1^ DW for ABA-GE; and 30 ng g^−1^ DW for neoPA.

### Cloning of full-length *Brassica ARF10* orthologs in *B. napus, B. oleracea* and *B. rapa*

Genomic clones harboring *Bna.ARF10* orthologs in Express 617 were isolated by hybridization to an 8x-coverage bacterial artificial chromosome (BAC) genomic library from Express 617. A deoxygenin-dUTP (DIG) labeled PCR amplicon from Express 617 was used a probe. The probe sequence, amplified by the PCR primer combination *ARF10* -ex1-1F/R (Table 1) corresponded to the conserved region between positions 416 to 600 in the *Arabidopsis thaliana ARF10* coding sequence (At2g28350, accession NM_128394). BAC filter hybridization and fluorescent detection was performed according to Garratt et al. (2001).

**Table 1:** **Primer sequences and optimum annealing temperatures used to amplify a conserved ***Brassica ARF10*** exonic region (ARF10-ex1-1) for BAC library screening, and full-length ***Brassica ARF10*** orthologs (BnARF10), respectively**.

**Primer name**	**Sequence**	**PCR annealing temperature (°C)**
ARF10-ex1-1F	5′-CGAGGCTTGATTACACGG-3′	54.4
ARF10-ex1-1R	5′-GCGGAGGAAGACGATTGA-3′	
BnARF10F	5′-AAAATGGAGCAAGAGAGAAG-3′	58.6
BnARF10R2	5′-ACAACCCAAACAAATAAAATT-3′	

A total of 70 positive BAC clones were identified. To isolate all full-length *Brassica* A and C genome orthologs of *ARF10*, 100 bp from the 5′- and 3′-ends of the *AtARF10* coding sequence were first blasted against *Brassica* genomic sequences available from the *Brassica* Genome Gateway at http://brassica.bbsrc.ac.uk/. Highly matching accessions were selected corresponding to the 5′ (CC952958, ES905909, EV092049) and the 3′ ends (BH703182, EX084979, EV067938, ES907226, BH605387) of *AtARF10*. Based on these *Brassica* sequences, a pair of consensus PCR primers was designed (Bn*ARF10* F/R2, see Table 1) to amplify full-length *Bna.ARF10* orthologs in the *Brassica* A and C genomes. The Bn*ARF10* F/R2 primers were also used to screen all 70 positive BAC clones. Five BAC clones harboring putative full-length *Bna.ARF10* sequences were selected for sequencing of the gene region. Nomenclature for the *Brassica* orthologs follows (Ostergaard and King, 2008).

The BnARF10F/R2 primers were also used to amplify full-length *ARF10* sequences from genomic DNA of *B. napus* 1012-98, *B. oleracea*, and B. rapa, respectively. All PCR reactions were carried out using PCR Extender proofreading polymerase enzyme (5 PRIME GmbH, Hamburg, Germany) in accordance with the manufacturer's guidelines. Full-length fragments were cloned into TOPO TA vector (Invitrogen, Darmstadt, Germany) for DNA sequencing. Bidirectional sequencing reactions were conducted by Eurofins MWG (Ebersberg, Germany). Express 617 sequences were generated directly from the six *Bna.ARF10*-positive BAC clones. Six randomly chosen clones were sequenced from full-length *Bna.ARF10* amplicons from 1012-98, along with four positive clones each from *B. oleracea* and B. rapa, respectively. Sequence and cluster analyses were performed using Vector NTI Advance 9.0, BioEdit 7.0.5, and CLC Sequence Viewer 6.4.

### Quantitative RT-PCR analysis for *B. napus Bna.ARF10, Bna.GH3.5*, and *Bna.GH3.6*

Total RNA from 100 mg freshly harvested seeds and total seedlings were isolated using TRIzol (Invitrogen). The protocol details can be referred to the work of MacRae (2007). The first-strand cDNA strand was synthesized from 5 μg DNA-free total RNA using RevertAid™ H Minus First Strand cDNA Synthesis Kit (Fermentas GmbH, St. Leon-Rot, Germany) and an oligo (dT) primer following the manufacturer's instructions. Diluted cDNA (1 μl) was used in 10 μl PCR containing 200 nM of each primer, 0.2 μl of ROX low and 5 μl KAPA SYBR® FAST (PEQLAB Biotechnologie GmbH, Erlangen, Germany). Three independent biological replicates were used for each sample, and quantification was performed in technical triplicate. PCR was performed in the 7500 fast RT-PCR (Applied Biosystems, Darmstadt, Germany) with the following temperature program: 10 min at 95°C, then 40 cycles of 15 s at 95°C, and 1 min at 60°C. At the end of the PCR, the melting temperature of the product was determined to verify the specificity of the amplified fragment. PCR product was analyzed using 7500 Software version 2.0.6 (Applied Biosystems, Darmstadt, Germany). The RT products of *Bna.ARF10, Bna.GH3.5*, and *Bna.GH3.6* were subjected to semi-quantitative PCR using BnaA+C.ARF10 forward (5′-GGRCAAGCKTTCGAAGTTGTTT-3′)/ BnaA+C.ARF10 reverse (5′-TCACGTCGGAAGCCTTCAC- 3′), BnaA+C.GH3.5 forward (5′- GGTGTGAACCTAAGGCCAC TTT-3′)/BnaA+C.GH3.5 reverse (5′-CGAAATAAGCCAT GGTCGGTAT-3′), and BnaA+C.GH3.6 forward (5′-TYTCACGCAATGCTGACGTT-3′)/BnaA+C.GH3.6 reverse (5′-WCTCRCGGTCGGTTCGT-3′) primers, respectively. Three classic housekeeping genes for vegetative stage in *Brassica* sp. namely β-actin (ACT2), tubulin (TUA), and ubiquitin (UBQ) were selected and analyzed using geNORM software package to identify the most stably expressed genes within a set of reference genes across three representative timepoints 4, 6, 8 DAS on the parental genotypes, Express 617 and 1012-98. For each reference gene, a stability value M was calculated; the lower the M-value the more stably the gene is expressed. ACT2 gene was selected as the best internal reference based on its stability in expression pattern in our test (data not show). Our test outcome is in agreement with Chandna et al. (2012) selection for the most suitable reference gene during vegetative stage in *Brassica juncea*. The relative expression levels of A- and C-copies of *B. napus ARF10* named *BnaA.ARF10.a.E617, BnaA.ARF10.b.E617, BnaA.ARF10.b.1012-98, BnaAC.ARF10.a.1012-98, BnaC.ARF10.a.E617, BnaC.ARF10.a.1012-98, BnaC.ARF10.b.E617, BnaC.ARF10.b.1012-98, BnaC.ARF10.c.1012-98*, and *BnaC.ARF10.d.1012-98 mRNAs* were calculated using the 2^−Δ*ΔCt*^ method normalized to the internal reference ACT2.

## Results

### Line 1012-98 has disturbed germination and seedling development

**Figure 1:**
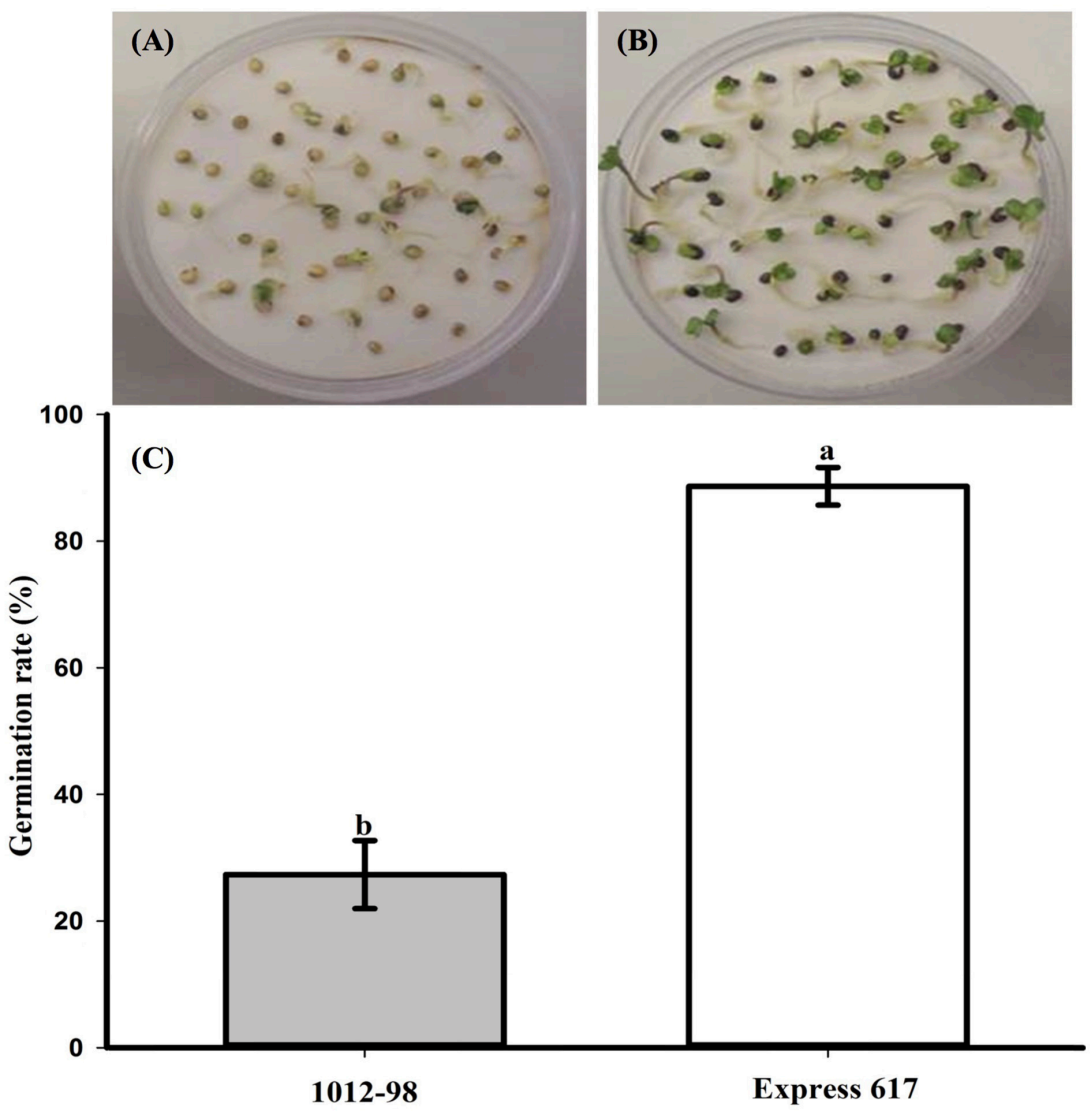
**Comparison of germination and germination rate (%) at 7 days after sowing in the phenotypically disturbed synthetic ***B. napus*** line 1012-98 (A, filled vertical bar C) and the normal line Express 617 (B, blank vertical bar C)**. Germination assessment was performed in Jacobsen chambers with 50 seeds per homozygous line under controlled conditions in a growth chamber. Values are presented as averages (± SEMs) with Student's *t*-test. Different letter superscripts between vertical bars indicate significant difference (*p* < 0.001).

**Figure 2:**
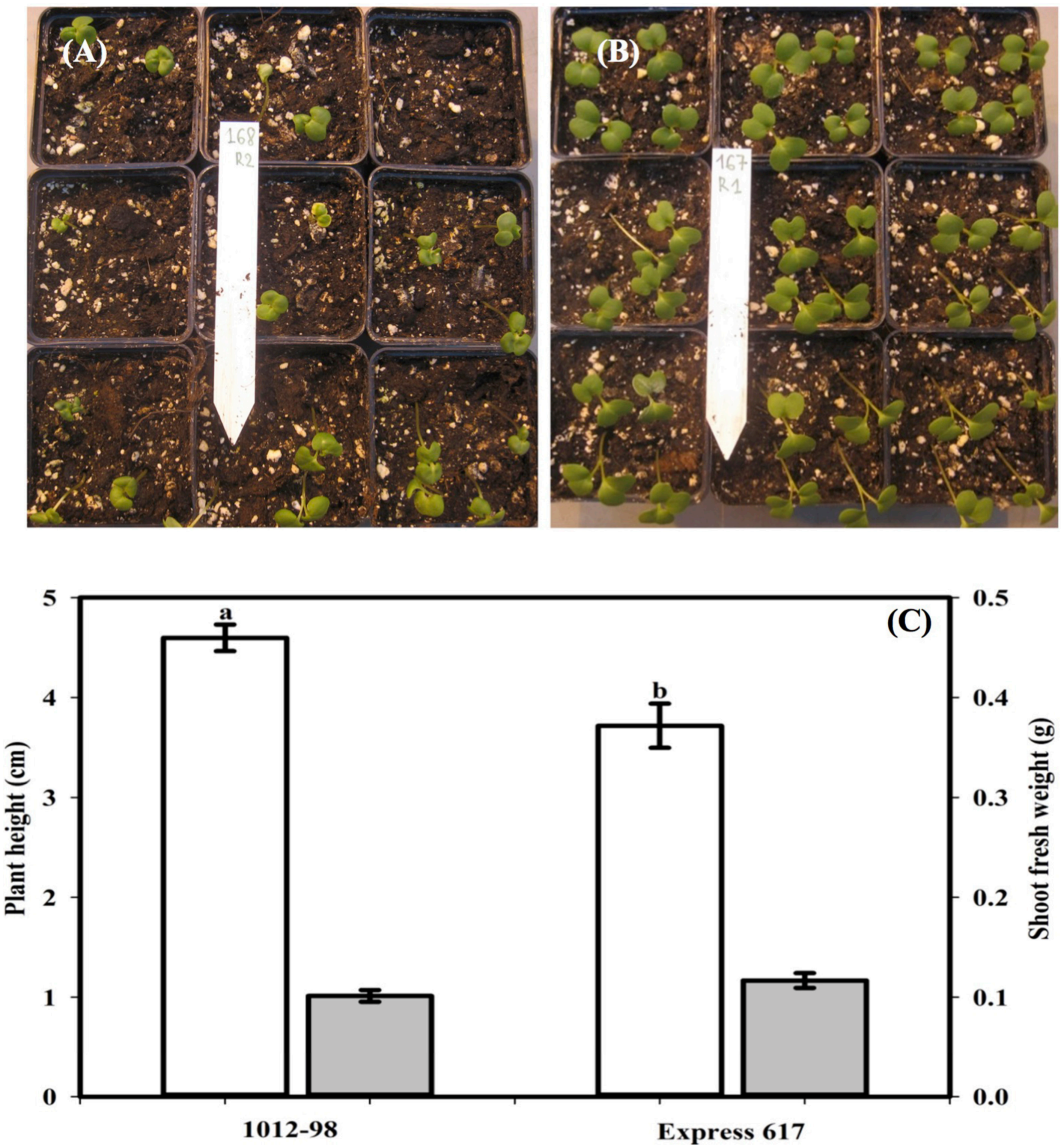
**Comparison of seedling vigor, plant height (cm, blank vertical bar), and shoot fresh weight (g, filled vertical bar) at 7 days after sowing in the phenotypically disturbed resynthesized ***B. napus*** line 1012-98 (A,C) and the normal line Express 617 (B,C)**. Seedling vigor was assessed in 36 soil-sown seeds per genotype grown under controlled conditions in a climate-controlled greenhouse. Values are presented as averages (±SEMs) with Student *t*-test. Different letter superscripts between vertical bars indicate significant difference (*p* < 0.05).

Figures 1, 2 compare the germination and seedling development in Express 617 and 1012-98. Whereas, Express 617 showed a normal germination rate of around 90%, the germination rate was severely depressed in 1012-98 with successful testa rupture and radicle emergence being observed in only around one quarter of the seeds (Figure 1C with ^***^p < 0.001). Express 617 also exhibited a considerably better seedling establishment than 1012-98 under greenhouse conditions. Seedlings of 1012-98 (Figure 2C) showed significantly increased etiolation and consequently longer hypocotyls than those of Express 617 (^*^p < 0.05) which was also found in transgene-mediated auxin overproduction in *Arabidopsis* (Romano et al., 1995). However, no significant difference was observed between the two genotypes for mean shoot fresh weight of successfully germinated plants at 7 DAS.

### Progenies from 1012-98 express abnormal developmental phenotypes

**Figure 3:**
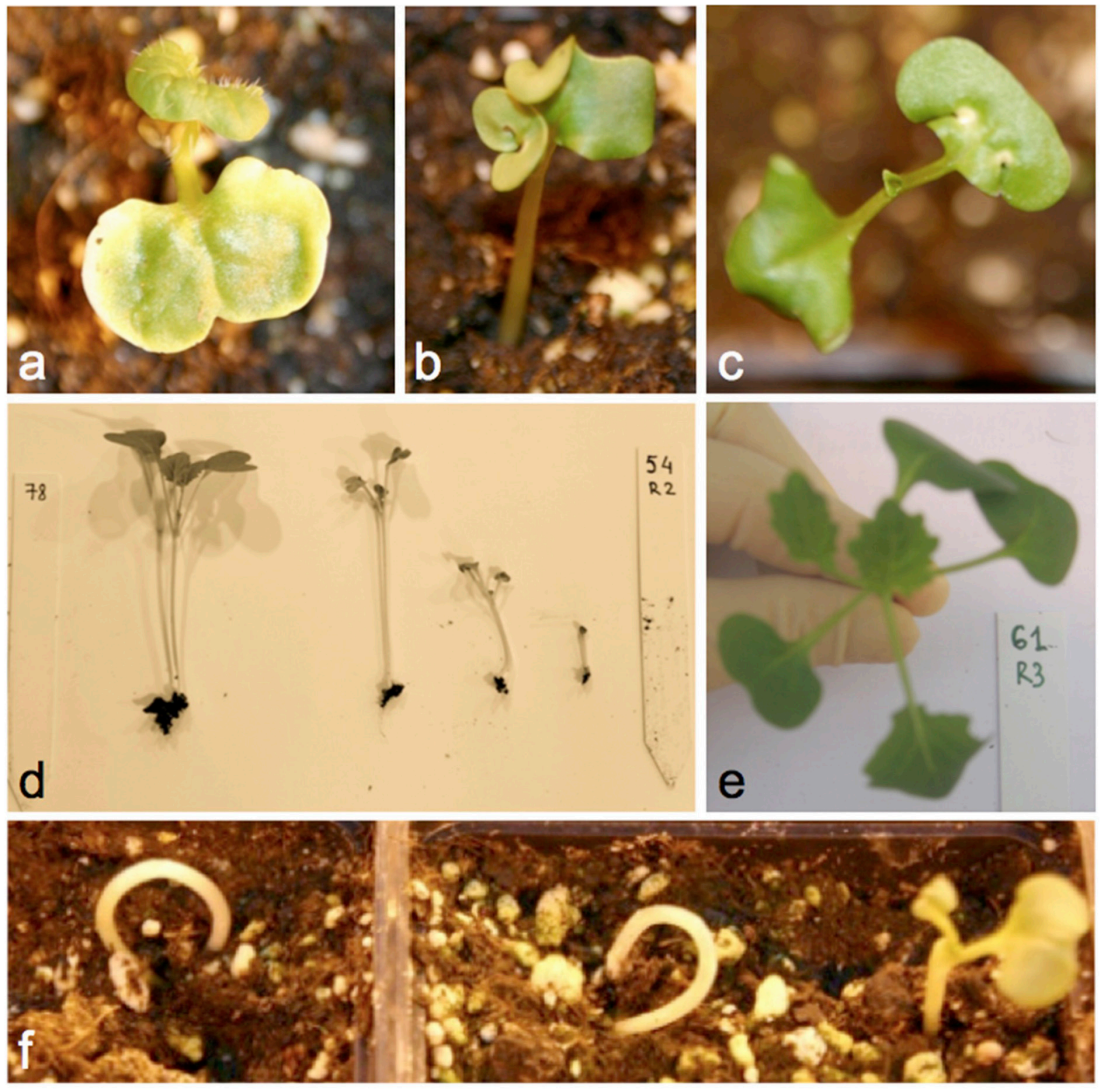
**Examples of commonly observed phenotypic abnormalities in seedlings of DH progenies from Express 617 × 1012-98 showing excessive IAA contents at 7 DAS. (A)** Ectopic trichromes, **(B)** deformed cotyledons, **(C)** chlorotic spots, **(D)** different degrees of stunted growth in the same homozygous line, **(E)** tricotyledons, **(F)** “ostrich” phenotype.

Figure 3 and Supplementary Table S2 give details of all documented seed and seedling phenotypes in 1012-98, Express 617, and their offspring, respectively. The DH progenies derived from the cross between 1012-98 and Express 617 showed a quantitative segregation for the germination defect and exhibited diverse developmental abnormalities during seedling development. No significant correlation was found between germination rate and seed color in the DH population, suggesting that these two traits are controlled by mutations at independent loci in the defective parent 1012-98. Abnormal developmental phenotypes included ectopic trichromes on cotyledons, curled cotyledons, tricotyledons, chlorotic spots on cotyledons, or chlorotic first leaves. Examples are shown in Figure 3. Seedlings with a weak hypocotyl and consequent “ostrich” phenotype, where the cotyledons failed to emerge from the soil, were common in the defective-development DH lines, as were seedlings with variable levels of stunted growth. Where the growth deformation was too severe the phenotype was often lethal. A high degree of variation was observed among individuals of the same homozygous genotype, with different degrees of developmental retardation being commonly observed within each hormone-defective line. This is typical for epigenetic variation and could indicate potential functional defects in one or more transcription factors involved in hormone-driven developmental regulation.

### 1012-98 seeds and seedlings show severe hormonal imbalance

**Figure 4:**
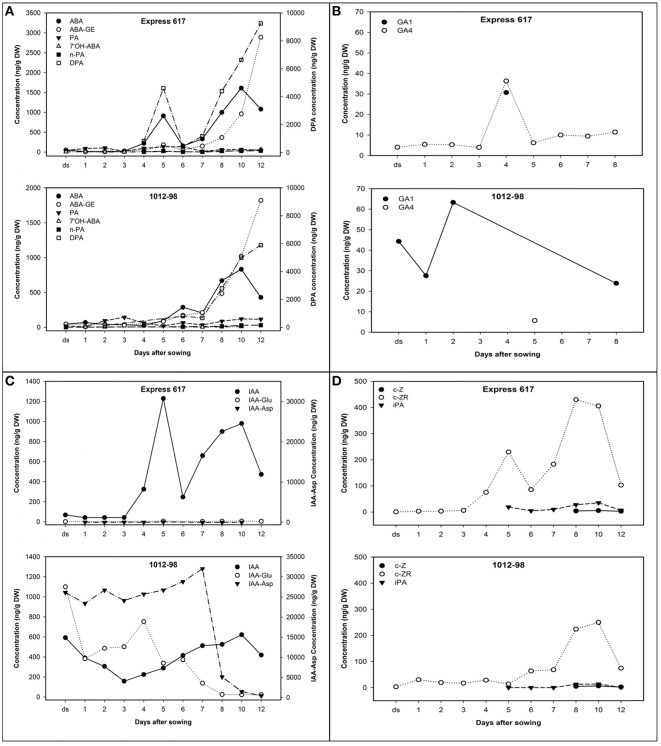
**Hormone profiles from dried seeds (ds) and germinating seedlings of Express 617 and 1012-98 after imbibition under 25°C, 55% RH and 16-h light. (A)** ABA and ABA metabolites, **(B)** GA metabolites, **(C)** auxin metabolites, and **(D)** cytokinins. The right-hand axes in **(A)** show the DPA concentrations, while the right-hand axes in **(C)** show concentrations of IAA-Aspartate (Asp). The first radicle protrusion appeared ~24 h after imbibition.

Hormone profiles of seeds and seedlings from 1012-98 and Express 617 until 12 DAS are described in Figure 4, with detailed data available in Supplementary Table S2. The desiccated, ripe seeds of the normal-germinating line Express 617 showed characteristically low levels of all major hormones and hormone metabolites. The dominant hormone in Express 617 was IAA, with a mean value of 68 ng/g dry weight (DW). In contrast, 1012-98 seeds contained ≈9-fold higher concentration of IAA and remained close to this proportion until 3 DAS, with an abnormally high level of 593 ng/g DW. IAA content in Express 617 declined gradually as compared to the sharply diminishing curve in 1012-98 within the first 3 DAS. The first 24 h after imbibition played a vital role in success of *B. napus* germination (Schopfer and Plachy, 1985). Despite of the remarkable reduction, IAA content in 1012-98 still remained 3.8-fold higher than the quantified amount in Express 617. The biggest difference in auxin profile between Express 617 and 1012-98 was extremely high levels of IAA-asp, IAA-glu and free IAA as late as 3 DAS on which is critical time for seed germination (Thakur and Sharma, 2016). An abnormally extreme concentration of more than 25,000 ng/g DW IAA-asp, higher than we have ever recorded in seeds of *Brassica* spp., increased to over 30,000 ng/g DW in 7 DAS before sharply declining to the lowest concentration of 454 ng/g DW at the end of study (see Supplementary Table S2).

The unusual levels of IAA conjugates were accompanied by uncharacteristically high seed GA levels in 1012-98. The only GA metabolites detected were GA_1_ and GA_4_, which in Express 617 appeared together in a brief burst around 4 DAS. In the normally germinating seeds of Express 617, the GA peak at 4 DAS preceded a sharp rise in IAA, DPA, ABA, and CK-ZR concentrations, at 5 DAS to support seedling growth. In 1012-98, on the other hand, abnormally high levels of GA_1_ were detected in the desiccated seeds and levels remained uncharacteristically high until the onset of post-germinative growth. Almost no GA_4_ was detected, with the exception of a minor peak at 5 DAS which presumably corresponds to the GA peak at 4 DAS in Express 617. This appears to be the deciding time-point that determines whether endosperm weakening and testa rupture is successful in 1012-98.

The dried seeds of both genotypes contained similar quantities of ABA. *De novo* synthesized ABA in 1012-98 started immediately within 24 h after imbibition with the concentration of 69.7 ng/g DW then decreased gradually until 4 DAS. The normal seeds of Express 617 produced ABA transiently at 5 DAS, however the ABA appeared to be rapidly inactivated through oxidation at the 8′-carbon atom, followed by rapid reduction of PA to DPA. DPA catabolism is not tracked in our study but likely occurs through conjugation to DPA glycoside. There is no evidence of significant conjugation of ABA at 5 days in Express 617. In both Express 617 and 1012-98 the ABA, DPA, and ABA-GE levels were found to rise after 6 days, with metabolism at this stage through both conjugation to the glucose ester and oxidation through the 8′-oxidation, with DPA pools rising and then falling.

In the DH progeny from Express 617 × 1012-98 the lines showing the most severe phenotypic abnormalities showed abnormally high levels of IAA conjugates, like those seen in 1012-98 (see Supplementary Table S2). This indicates that the developmental phenotypes are heritable and associated with a genetically determined hormonal imbalance derived from 1012-98. The observed phenotypes are consistent with auxin-overdose symptoms, as described by Boerjan et al. (1995) and Romano et al. (1995).

### Copy number amplification and intergenomic recombination in *Bna.ARF10* orthologs

Each of the four sequenced *ARF10* clones from *B. rapa* and *B. oleracea*, respectively, gave identical sequences (hereinafter designated *BraA.ARF10* and *BolC.ARF10*, respectively). This indicates either a single locus of this gene in each of the two diploid species, or complete identity of paralogs in the respective genomes, which are each considered to be ancestral tetraploids (Lysak et al., 2005).

**Figure 5:**
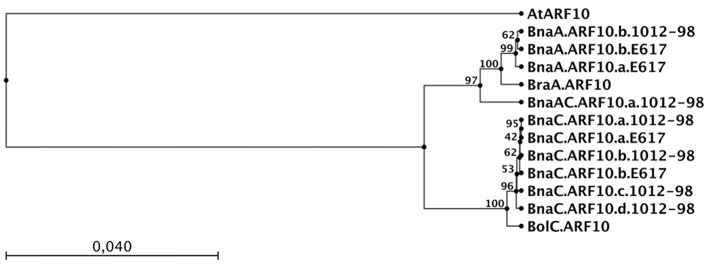
**UPGMA tree showing the relationship between genomic coding DNA sequences of ***ARF10*** orthologs from ***A. thaliana*** (At), ***B. napus*** (Bna) Express 617 (E617) and 1012-98, ***B. rapa*** (Bra) and ***B. oleracea*** (Bol)**. The capital letter following the 3-letter *Brassica* species code represents the A or C genome origin of the sequence. The tree was constructed using CLC Sequence Viewer 6.4. Arm lengths represent average genetic distances between clusters. Bootstrap values from 100 repetitions of the UPGMA cluster are shown on the nodes.

The sequences we obtained from the amphidiploid *B. napus* were derived from homozygous inbred lines, hence each unique sequence represents an independent, homeologous *ARF10* locus. Two distinct loci with very high homology to *BraA.ARF10* were found (hereinafter referred to as *BnaA.ARF10*.a and *BnaA.ARF10*.b), suggesting a duplication event in the *B. napus* A genome following polyploidization. Interestingly, in place of *BnaA.ARF10.a* the resynthesized *B. napus* line 1012-98 contained a novel recombinant allele, here designated as *BnaAC.ARF10.a*.*1012-98*, that was derived from a concatenation of exon 1 from the *BraA.ARF10* locus with exons 2, 3, 4 from *BnaC.ARF10*. This recombinant allele likely resulted from a non-homeologous recombination event, a frequent phenomenon during the first meiosis of resynthesized *B. napus* genotypes (Udall et al., 2005; Szadkowski et al., 2010). In the UPGMA tree (Figure 5) the *BnaAC.ARF10.a*.*1012-98* sequence was found to cluster at an intermediate position between the A and C genome *ARF10* sequences, reflecting its origin as a chimeric locus.

Express 617 and 1012-98 shared two common C genome *ARF10* loci (hereinafter referred to as *BnaC.ARF10.a* and *BnaC.ARF10.b*), however in 1012-98 we unexpectedly detected two additional C-genome loci (designated here as *BnaC.ARF10.c* and *BnaC.ARF10.d*). As in the A genome, the C genome *ARF10* locus from *B. oleracea* also appears to have been duplicated during the evolution of natural *B. napus* (Express 617), while the resynthesized *B. napus* line 1012-98 shows additional copy number amplification from two to four C genome *ARF10* copies. Alignment of all detected *Bna.ARF10* copies against *Brassica* ESTs showed that all copies present in both Express 617 and 1012-98 have very high homology to expressed transcripts, and all have over 99% similarity to *BolC.ARF10*. This suggests that all C-genome copies could be potentially transcriptionally active. Therefore, the quadruplication event in 1012-98 might be expected to increase *ARF10* transcript abundance, thus causing an over-accumulation of m*ARF10* in auxin signal transduction throughout plant growth development. Liu et al. (2007) found that repression of *ARF10* is essential for normal seed germination and seedling establishment in *A. thaliana*, hence such an over-accumulation is expected to have a negative influence on these processes.

### Strong evolutionary conservation of *Brassica* ARF10 protein domains

An overview of predicted structural parameters of all identified *Brassica ARF10* orthologs is given in Table 2, while Supplementary Table S3 compares pairwise DNA and deduced protein sequence identities among all detected *Brassica ARF10* loci and *AtARF10*. A-genome alleles aligned with *BnaA07g13830D* gene (≥99% similarity) on chromosome A07 while C-genome alleles matched to BnaC04g15900D (≥98.7% similarity) gene chromosome C04 (Chalhoub et al., 2014). The complete amino acid sequences for all investigated loci are provided in Figure 1. The genomic and coding sequences of *BraA.ARF10* and *BolC.ARF10* were 97% identical, while the deduced protein alignments revealed even higher conservation at the protein level than at the nucleotide level. Protein sequence conservation of over 99% was observed within the respective A-genome and C-genome loci, with over 98% conservation observed between A and C genome loci in B. rapa, *B. oleracea* and *B. napus*, respectively. The *Brassica ARF10* genomic sequences showed 85% identity to the corresponding genomic sequence of *AtARF10*, with 88% identity among the coding sequences. Comparisons with the NCBI Conserved Domains Database (CDD) revealed that the predicted proteins from all *Brassica ARF10* orthologs share the same three highly-conserved domains with *AtARF10*: a conserved auxin responsive factor, a B3-DNA binding domain, and an AUX/IAA super-family domain.

**Table 2:** **Predicted parameters of ***ARF10*** products from ***A. thaliana*** (***At***), ***B. oleracea*** (***Bol***), ***B. rapa*** (***Bra***), ***B. napus*** (***Bna***) Express 617 (E617), and ***B. napus*** 1012-98, respectively**.

**Locus**	**Origin of allele**	**Accession number**	**Chromosome/Identity (%)**	***B. napus* genes**	**Amino acids**	**MW (kDa)**	**pI**	**Conserved domains (NCBI CDD)**		
								**B3 DNA binding domain**	**Auxin response factor**	**AUX/IAA family**
*AtARF10*	-	At2g28350	2		693	76.7	7.71	F_115_-A_216_	G_284_-Q_360_	S_574_-I_656_
*BraA.ARF10*	-	JX494287	A07/99	BnaA07g13830D	705	77.9	7.33	F_112_-A_213_	G_288_-Q_364_	G_586_-I_668_
*BolC.ARF10*	-	JX494286	C04/99.5	BnaC04g15900D	703	77.9	7.04	F_112_-A_213_	G_287_-Q_363_	G_584_-I_666_
*BnaA.ARF10.a*	E617	JX456096	A07/99	BnaA07g13830D	705	77.9	8.00	F_112_-A_213_	G_288_-Q_364_	G_586_-I_668_
*BnaA.ARF10.b*	E617	JX456097	A07/99.3	BnaA07g13830D	706	78.0	8.00	F_112_-A_213_	G_288_-Q_364_	G_587_-I_669_
	1012-98	JX456098	A07/99.1	BnaA07g13830D	706	78.0	8.00	F_112_-A_213_	G_288_-Q_364_	G_587_-I_669_
*BnaAC.ARF10.a*	1012-98	JX456089	C04/98.7	BnaC04g15900D	704	77.8	7.02	F_112_-A_213_	G_288_-Q_364_	G_585_-I_667_
*BnaC.ARF10.a*	E617	JX456090	C04/100	BnaC04g15900D	703	77.8	6.59	F_112_-A_213_	G_287_-Q_363_	G_584_-I_666_
	1012-98	JX456092	C04/100	BnaC04g15900D	703	77.8	6.59	F_112_-A_213_	G_287_-Q_363_	G_584_-I_666_
*BnaC.ARF10.b*	E617	JX456091	C04/100	BnaC04g15900D	703	77.8	6.59	F_112_-A_213_	G_287_-Q_363_	G_584_-I_666_
	1012-98	JX456093	C04/99.9	BnaC04g15900D	703	77.8	6.59	F_112_-A_213_	G_287_-Q_363_	G_584_-I_666_
*BnaC.ARF10.c*	1012-98	JX456094	C04/100	BnaC04g15900D	703	77.8	6.59	F_112_-A_213_	G_287_-Q_363_	G_584_-I_666_
*BnaC.ARF10.d*	1012-98	JX456095	C04/99.9	BnaC04g15900D	703	77.8	6.79	F_112_-A_213_	G_287_-Q_363_	G_584_-I_666_

*The locus nomenclature is explained in the legend to Table 3*.

### 1012-98 has amino-acid substitutions in *BnaC.ARF10* DNA-Binding domains

All *Brassica ARF10* sequences were submitted to NCBI with the accession numbers presented in Table 2. Frequent functional mutations compared to *A. thaliana, B. rapa* and *B. oleracea* were observed in *B. napus* A-genome *ARF10* sequences from both the mutant 1012-98 and also the normal line Express 617. This suggests that the C-genome homeologs of this gene family probably have more functional relevance in *B. napus* than the two A-genome homeologs. Besides the four native C-genome *ARF10* copies in 1012-98 (compared with only two in Express 617), the chimeric AC sequence that was found in 1012-98 in place of *BnaA.ARF10.a* also contains the DNA-binding domain (DBD) from the C-genome donor. We hypothesize that this substantial increase in copy number, from two to five *Bna*.*ARF10* copies with C-genome DBDs, strengthens repression of auxin response genes in 1012-98, leading to phenotypes that closely mimic the mi160 resistance described in *Arabidopsis* by (Liu et al., 2007).

Comparison of *Bna.ARF10* nucleotide sequences from Express 617 and 1012-98 to the *Brassica ARF10* consensus sequence (Table 3) revealed a total of eight SNPs in 1012-98 alleles from *BnaC.ARF10* loci, seven of which appear within the highly conserved DNA binding domains. Three SNPs in the AUX/IAA family domains of *BnaC.ARF10.b* and *BnaC.ARF10*.d do not result in amino acid substitutions, while a fourth is a putative non-functional A > G substitution in intron 4. In contrast, two SNPs in 1012-98 result in amino acid substitutions, one in the B3 DNA-binding domain (W162 to R162) and one in the ARF domain (S333 to L333), respectively. Express 617 exhibited no SNPs of putative functional relevance: An A > G substitution was observed in intron 4, a synonymous T > C substitution at C593 in the AUX/IAA family domain of *BnaC.ARF10.b* and three SNPs outside the functional domains of the A-genome allele *BnaA.ARF10.a*.

**Table 3:** **Detected SNPs (underlined) and corresponding amino acids (bold) in ***Bna.ARF10*** alleles from Express 617 and 1012-98 compared to the ***Brassica ARF10*** consensus sequence (see also Supplementary Figure S1)**.

**Locus/allele^a^**	**Position in genomic/coding sequence**	**Codon/amino acid substitutions**	**Predicted functional relevance**
*BnaA.ARF10.a.E617*	324/324	AAA/**K_108_** → AAG/**K_108_**	
*BnaC.ARF10.d.1012-98*	484/484	TGG/**W_162_** → CGG/**R_162_**	B3 DNA binding domain
*BnaC.ARF10.b.1012-98*	861/861	GGA/**G_287_** → GGG/**G_287_**	Auxin response factor
*BnaC.ARF10.c.1012-98*	930/930	GTG/**V_310_** → GTA/**V_310_**	Auxin response factor
*BnaC.ARF10.d.1012-98*	1001/1001	TCG/**S_333_** → TTG/**L_333_**	Auxin response factor
*BnaA.ARF10.a.E617*	1213/1136	GTT/**V_379_** → GCT/**A_379_**	
*BnaA.ARF10.a.E617*	1569/1492	TCC/**S_498_** → CCC/**P_498_**	
*BnaC.ARF10.b.E617*	1863/1779	TGT/**C_593_** → TGC/**C_593_**	AUX/IAA family
*BnaC.ARF10.b.1012-98*	1869/1785	GTT/**V_595_** → GTC/**V_596_**	AUX/IAA family
*BnaC.ARF10.b.1012-98*	1890/1806	GTT/**V_602_** → GTA/**V_602_**	AUX/IAA family
*BnaC.ARF10.d.1012-98*	2019/1935	GTT/**V_645_** → GTC/**V_645_**	AUX/IAA family
*BnaC.ARF10.b.E617*	2067 (intron 4)	A → G	
*BnaC.ARF10.a.1012-98*	2231 (intron 4)	A → G	

a *Locus/allele nomenclature follows the convention for Brassica spp. described by Ostergaard and King (2008): [Species—3 letter code; genome—A or C; gene—ARF10]. [locus—a, b, c or d]. [allele—in this case E617 or 1012-98]*.

### High accumulation of active IAA and its metabolites in 1012-98 caused minor and major up-regulations of *Bna.ARF10* and *Bna.GH3.5*, respectively

**Figure 6:**
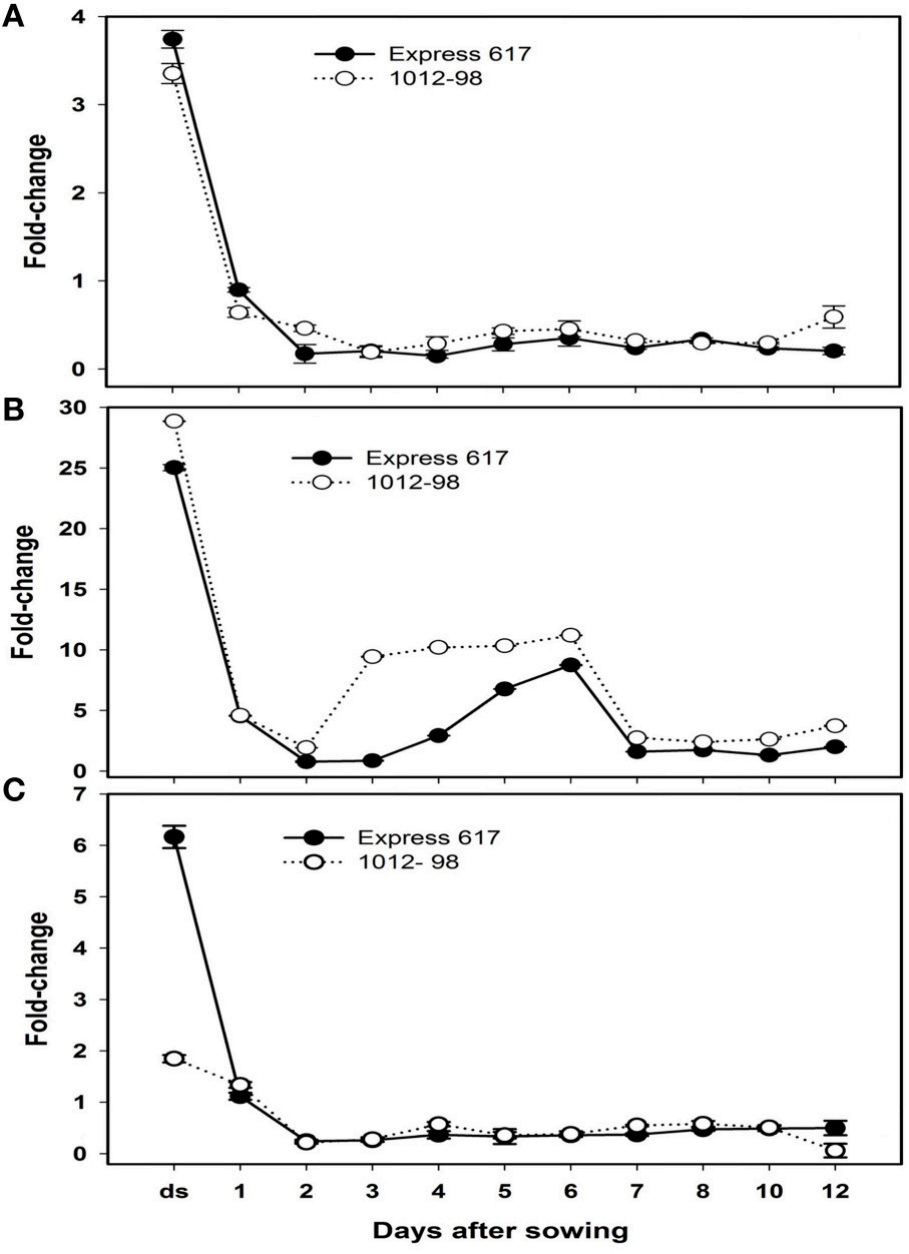
**Quantitative real-time PCR analysis of (A) ***Bna.ARF10***, (B) ***Bna.GH3.5***,and (C) ***Bna.GH3.6*** expression in Express 617 and 1012-98**. Results were normalized against the expression of ACT2. Error bars denote SEMs.

To learn about the differential expression of *Bna.ARF10, Bna.GH3.5*, and *Bna.GH3.6* between Express 617 and 1012-98 in response to auxin signaling, quantitative PCR was used with the specific primers BnaA+C.*ARF10* forward/reverse to amplify both A- and C-genome of *Bna.ARF10, Bna.GH3.5*, and *Bna.GH3.6* genes from 0 to 12 DAS. Both *Bna.ARF10* and *Bna.GH3.5* highly expressed in dried seed, declined rapidly within 24 h after imbibition and remained close to these levels in both genotypes to the end of this study (Figures 6A,B). Expression level of *Bna.ARF10* in 2 and 12 DAS was ~2.7-fold higher in 1012-98 than in Express 617. Coupling of up-regulation *Bna.ARF10, Bna.GH3.5* transcripts in 1012-98 was 2.5-, 11-, and 3.5-fold higher on 2, 3, and 4 DAS as compared with those in Express 617, respectively. These upregulations of *Bna.GH3.5* corresponded to the reduction of active IAA and the increase of IAA-asp between 1 and 4 DAS. In contrast, *Bna.GH3.6* expression in ripe seeds and 12 DAS accumulated 3.3- and 8.1-fold higher in Express 617 than in 1012-98, respectively while non-significant difference in transcription levels was observed between two genotypes (Figure 6C).

## Discussion

Seed germination is controlled by genetics and the crosstalk of hormonal components mainly ABA and GA (Holdsworth et al., 2008). Recent studies have provided the evidence of auxin involvement in both germination completion and seedling establishment by increasing seed sensitivity to ABA (Ogawa et al., 2003; Liu et al., 2007). the present study, seeds of the mutant *B. napus* line 1012-98 produced higher content of ABA and IAA as late as 3 DAS, the critical timepoint for successful germination and emergence into post-germinative growth (Thakur and Sharma, 2016). Furthermore, 1012-98 and its hormone-defect offspring showed severe ABA-hypersensitivity phenotypes, and there was no evidence in 1012-98 during the first few days after imbibition for the necessary catabolism of ABA to less active PA and its inactive form, DPA. We found that the decline of ABA after imbibition in normal *B. napus* was accompanied by an increase in the level of less active phaseic acid (PA) conjugates, coinciding with the transition from germination to post-germinative growth. This suggests that 8′-OH ABA was formed first and then cyclized to form PA. This kind of ABA catabolism has been shown to be important in regulating germination potential (Kushiro et al., 2004; Okamoto et al., 2006). The high accumulation of free ABA in 1012-98 at 3 DAS was probably derived from *de novo* ABA biosynthesis, which is believed to play a key role in dormancy maintenance or delay of germination (Kushiro et al., 2004). In contrast, strong *de novo* synthesis of ABA in Express 617 occurred only post-germination.

ABA catabolism is primarily driven by 8′-hydroxylation encoded by the CYP707A gene family. The product of hydroxylation, 8′-hydroxy ABA, is converted to PA and subsequently inactivated as DPA (Nambara and Marion-Poll, 2005). The ABA decline at 5 DAS in *B. napus* Express 617 was not accompanied by an increase in concentration of its metabolites and/or conjugates, besides a very small accumulation of PA. On the other hand, the decrease in ABA content at 10 DAS coincided with 3-fold increase of ABA-GE. This suggests two independent ABA catabolic pathways, one fluxed through PA in the germinative phase and the other involved independently in post-germinative development.

### Extreme auxin excess inhibits germination in *B. napus* 1012-98

Inhibition of germination by IAA was well-documented in lettuce over 50 years ago (Khan and Tolbert, 1966). In fact, auxin inhibits seed germination by enhancing ABA action driven by transcription factor ABSCISIC ACID INSENSITIVE 3 (*ABI3*), thereby adding secondary protective level of control in the regulation of seed dormancy and germination. Auxin action in *Arabidopsis* seed germination requires the ABA signaling and the roles of auxin-ABA in seed dormancy are interdependent (Liu et al., 2013). The concentration of IAA and its metabolites in 1012-98 was remarkably high in dried seed and as late as 3 DAS on which success or failure of seed germination were determined. Additionally, IAA-asp remained at extreme levels until the transition into post-germination development. The high accumulation of endogenous auxin and ABA (over 4-fold higher) within 48 h after imbibition, these conditions probably created an ABA-hypersensitive phenotype in yellow-seeded 1012-98 line, thereby the seeds were failed to protrude radicles under favorable conditions. Additionally, successfully germinated seedlings of 1012-98 expressed significantly elongated hypocotyls under post-germinative auxin action (Figure 2). In *Arabidopsis*, both auxin-overproducing transgenic line *iaaM-OX* and exogenous auxin applied wild-type seeds did not germinate up to 30 DAS in germination assay due to inhibitory effect of ABA in a dose-dependent manner (Liu et al., 2007, 2013). Taken together, these results explains the extremely poor germination and seedling establishment observed in the *B. napus* mutant 1012-98, in which the characteristic IAA pattern was completely disturbed.

### 1012-98 phenotypes are consistent with endogenous IAA overdose

Auxin acts upstream of major regulator *ARF10*. Additionally, repression of *ARF10* by MIR160 is critical for seed germination and post-germination stages (Liu et al., 2007). The mutant phenotypes we observed in germinated 1012-98 seedlings and its hormone-defective offspring (Figure 3) correspond strikingly to those of *Arabidopsis* auxin overproduction or *arf10* mutants, which show similar developmental defects including elongated hypocotyl, small, and epinastic/deformed cotyledons, tricotyledon, reduced number of leaves, constricted apical end of hypocotyl, and curled stems (Boerjan et al., 1995; Romano et al., 1995; Liu et al., 2007). Disturbed apical hook development, causing the “ostrich” phenotypes we observed in 1012-98 and its offspring, has been most extensively connected with auxin. A common cause is auxin inhibition of cell expansion at the inner side GA promotion of cell division and expansion at the outer side of the hook (Vandenbussche et al., 2010; Mazzella et al., 2014). Stem curling and leaf malformation are also among the first tri-phasic responses of plants after application of the synthetic auxin herbicide 2, 4-D, followed by stunted growth, chlorosis, wilting, necrosis, and ultimately death. Excessive concentrations of endogenous IAA cause an imbalance in auxin homeostasis and in interactions with ABA and ethylene. Notably, the deformative and growth-inhibiting effects caused by application of synthetic auxin are also observed in IAA-overproducing transgenic plants (Grossmann, 2010). Irregular distribution of auxin leading to disturbed cell elongation is the most likely explanation for the hook-like seedlings, stem curvature, twisted petioles and other developmental phenotypes observed in Express 617 × 1012-98 DH lines despite successful germination.

### The hypersensitive-to-ABA mutant 1012-98 lacks sufficient GA for successful germination

The balance of endogenous ABA and GA levels serves an important role in controlling seed germination (Finch-Savage and Leubner-Metzger, 2006). GA_1_ levels in 1012-98 were unusually high in comparison with Express 617. In contrast the levels of GA_4_, which in *Arabidopsis* seeds is around 10 times more active than GA_1_ (Yang et al., 1995), were not detected until 5 DAS in the *B. napus* mutant. In the context of excess ABA content and extremely high auxin accumulation, the mutant 1012-98 genotype did not produce sufficient amount of active GAs to deactivate the ABA inhibition and complete germination. In fact, germination of 7B-1 ABA-hypersensitive mutant tomato line was able to restore with application of exogenous GA_3_ (Fellner et al., 2001). The hormone profile of 1012-98 suggests that elevated levels of GA would be required to successfully promote germination in the presence of excessive ABA. In Express 617, the accumulation of free auxin and ZR at 5 and 10 DAS, respectively, appeared sufficient to successfully induce the transition from germination to post-germinative growth.

### Molecular links of transcriptional repressor *Bna.ARF10* to auxin-response genes *Bna.GH3.5, Bna.GH3.6*, and high auxin accumulations

*ARF10* is important for several aspects of plant growth and development, at least some of which appear to be dependent on GH3 gene family (Mallory et al., 2005; Guilfoyle and Hagen, 2007). Recently the crosstalk between auxin and ABA signaling in controlling *Arabidopsis* seed dormancy and germination have been proven and ABA function is largely dependent on the TIR1/AFB-AUX/IAA-ARF-mediated auxin signaling pathway. In fact, auxin acts upstream of the major regulator of seed dormancy, *ABI3*, by recruiting the *ARF10* and *ARF16* to control the *ABI3* expression during seed germination (Liu et al., 2007, 2013). In *Arabidopsis*, both *ARF10* and *ARF17* are classified as transcriptional repressors with an enrichment in serine (S), proline (P), leucine (L), and either glycine (G; ARF10), or theronine (T; ARF17) residues (Tiwari et al., 2003). The deduced protein sequences in *Brassica* in this study confirmed SPL-rich regions located just before and after the conserved domains. On the background of high accumulation of ABA and auxin as late as 3 DAS in yellow-seeded 1012-98 genotype, the copy number amplification of *Bna.ARF10* in 1012-98 leading to minor transcriptional increase presumably added another restriction on germinative process through the inhibitory action of *ABI3*. The upregulation of *Bna.GH3.5* between 2 and 6 DAS in ABA-hypersensitive 1012-98 corresponded to the second peak (4 DAS) of IAA-asp and the reduction of active IAA (3 DAS). In fact, the *GH3*-like gene family plays an essential role in maintaining optimal levels of endogenous IAA through the amino acid, sugar, and peptide-linked conjugating pathways in plant cells (Woodward and Bartel, 2005). Besides the previously described copy number variation for *BnaC.ARF10*, the major functionally relevant *Bna.ARF10* sequence divergence associated with the developmental disruption in 1012-98 appears to be present in *BnaC.ARF10*.d. Mutations in the B3 D NA-binding domains might enhance binding activities of *BnaC.ARF10*.d to *AuxRE* and to *Aux/IAA* proteins in 1012-98. Taken together, this can be expected to lead to the germinative hyper-repressor in 1012-98, which in turn would cause the very poor germination and disrupted transition from germination to seedling development. It can be proposed that the crosstalk of auxin-ABA might have coevolved to synergically control seed dormancy to survive unfavorable environment. These mutations appear to have arisen independently during the *de novo* allopolyploidization of 1012-98 from an interspecific cross between *B. rapa* and *B. oleracea*.

## Author contributions

TN designed, carried out research and wrote the manuscript. SA analyzed seedling metabolomics. CO designed the quantitative RT-PCR experiments. RS and WF participated in study design and revised the manuscript. All authors read and approved the final manuscript.

### Conflict of interest statement

The authors declare that the research was conducted in the absence of any commercial or financial relationships that could be construed as a potential conflict of interest.

## Supplementary material

Supplementary Figure S1 **Multi-alignment of ***ARF10*** proteins from ***Arabidopsis thaliana*** (At), ***Brassica rapa*** (Bra), ***B. oleracea*** (Bol), and the ***B. napus*** (Bna) lines Express 617 (E617) and 1012-98**. The background shading represents the degree of similarity, with 100% conserved amino acids shown by black shading, strongly conserved amino acids in gray tones and variable positions in white. Locus/allele nomenclature follows the convention for *Brassica* spp. described by Østergaard and King (2008): [Species—3 letter code; genome—A or C]. [gene—*ARF10*; locus]. [allele—in this case E617 or 1012-98].

Supplementary Table S1 **Internal standards used for hormone quantification by HPLC-ESI-MS/MS**.

Supplementary Table S2 **See and seedling phenotype data for 1012-98, Express 617 and 166 doubled haploid (DH) lines derived from their F_**1**_ hybrid**.

Supplementary Table S3 **Pairwise identities (%) in genomic/coding regions (above diagonal) and deduced protein sequences (below diagonal) from ***ARF10*** orthologs in ***A. thaliana*** (At), ***B. rapa*** (Bra), ***B. oleracea*** (Bol) natural ***B. napus*** (***Bna***) line Express 617 (E617) and resynthesized ***B. napus*** line 1012-98, respectively**. Locus/allele nomenclature follows the convention for *Brassica* spp. described by Østergaard and King (2008): [Species—3 letter code; genome—A or C; gene—*ARF10*]. [locus]. [allele—in this case E617 or 1012–98].
