# Supplementary material for: Disruption of Germination and Seedling Development in Brassica napus by Mutations Causing Severe Seed Hormonal Imbalance
Source: Front Plant Sci. 2016 Mar 15;7:322. doi: 10.3389/fpls.2016.00322 (PMC4791391; doi:10.3389/fpls.2016.00322)
Supplement: Supplementary Figure S1 — Multi-alignment of ARF10 proteins from Arabidopsis thaliana (At), Brassica rapa (Bra), B. oleracea (Bol), and the B. napus (Bna) lines Express 617 (E617) and 1012-98. The background shading represents the degree of similarity, with 100% conserved amino acids shown by black shading, strongly conserved amino acids in gray tones and variable positions in white. Locus/allele nomenclature follows the convention for Brassica spp. described by Østergaard and King (2008): [Species—3 letter code; genome—A or C]. [gene—ARF10; locus]. [allele—in this case E617 or 1012-98]. [file Image1.PDF]

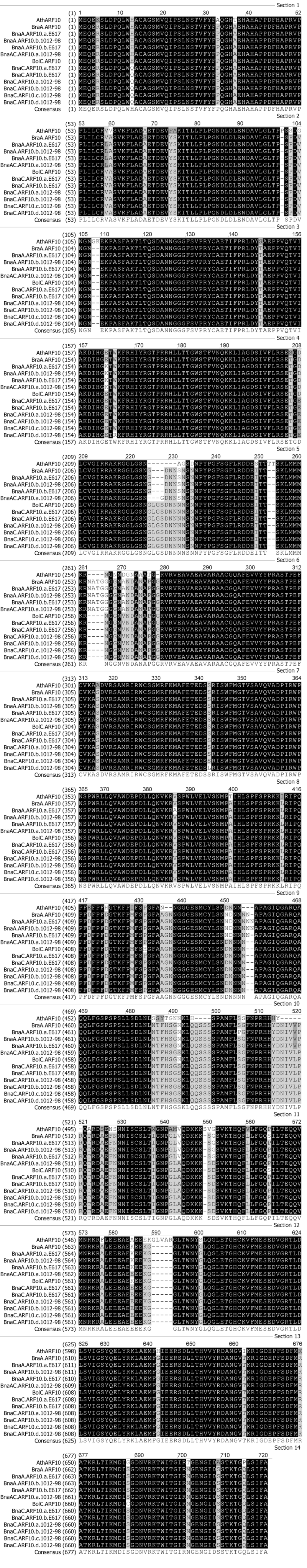

Section 1
Section 2
Section 3
Section 4
Section 5
Section 6
Section 7
Section 8
Section 9
Section 10
Section 11
Section 12
Section 13
Section 14
AthARF10
BraA.ARF10
BnaA.ARF10.a.E617
BnaA.ARF10.b.1012-98
BnaA.ARF10.b.E617
BnaA.ARF10.a.1012-98
BolC.ARF10
BnaC.ARF10.a.E617
BnaC.ARF10.b.E617
BnaC.ARF10.a.1012-98
BnaC.ARF10.b.1012-98
BnaC.ARF10.c.1012-98
BnaC.ARF10.d.1012-98
Consensus
