# Supplementary material for: Disruption of Germination and Seedling Development in Brassica napus by Mutations Causing Severe Seed Hormonal Imbalance
Source: Front Plant Sci. 2016 Mar 15;7:322. doi: 10.3389/fpls.2016.00322 (PMC4791391; doi:10.3389/fpls.2016.00322)
Supplement: Supplementary Table S1 — Internal standards used for hormone quantification by HPLC-ESI-MS/MS. [file Table1.PDF]

Supplementary Table S1.

Internal standards used for hormone quantification by HPLC-ESI-MS/MS.

Metabolite	Abbreviation	Source
Abscisic acid	ABA	Sigma-Aldrich, St. Louis, MO, USA
Indole-3-acetic acid	IAA	
Indole-3-acetic acid aspartate	IAA-asp	
Zeatin	Z	
Zeatin riboside	ZR	
Isopentenyl adenosine	iPA	
Isopentenyl adenine	2iP	
Dihydrozeatin	dhZ	Olchemim Ltd., Olomouc, Czech Republic
Dihydrozeatin riboside	dhZR	
Zeatin-O-glucoside	Z-O-Glu	
Duterated forms	d3-DPA	NRC-PBI, Saskatoon, SK, Canada
	d5-ABA-GE	
	d3-PA	
	d4-70-OHABA	
	d3-neoPA	
	d4-ABA	
	d3-IAA-Asp	
	d3-IAA-Glu	
	d5-IAA	Cambridge Isotope Laboratories, Andover, MA, USA
	d3-dhZ	Olchemim Ltd., Olomouc, Czech Republic
	d3-dhZR	
	d5-Z-O-Glu	
	d6-iPA	
	d6-2iP	
Recovery standards	d6-ABA	NRC-PBI, Saskatoon, SK, Canada
	d2-ABA-GE	
