# Supplementary material for: Disruption of Germination and Seedling Development in Brassica napus by Mutations Causing Severe Seed Hormonal Imbalance
Source: Front Plant Sci. 2016 Mar 15;7:322. doi: 10.3389/fpls.2016.00322 (PMC4791391; doi:10.3389/fpls.2016.00322)
Supplement: Supplementary Table S3 — Pairwise identities (%) in genomic/coding regions (above diagonal) and deduced protein sequences (below diagonal) from ARF10 orthologs in A. thaliana (At), B. rapa (Bra), B. oleracea (Bol) natural B. napus (Bna) line Express 617 (E617) and resynthesized B. napus line 1012-98, respectively. Locus/allele nomenclature follows the convention for Brassica spp. described by Østergaard and King (2008): [Species—3 letter code; genome—A or C; gene—ARF10]. [locus]. [allele—in this case E617 or 1012–98]. [file Table3.PDF]

Supplementary Table S3. Pairwise identities (%) in genomic/coding regions (above diagonal) and deduced protein sequences (below diagonal) from *ARF10* orthologues in *A. thaliana* (*At*), *B. rapa* (*Bra*), *B. oleracea* (*Bol*) natural *B. napus* (*Bna*) line Express 617 (E617) and resynthesised *B. napus* line 1012-98, respectively. Locus/allele nomenclature follows the convention for *Brassica* spp. described by Østergaard and King (2008): [Species – 3 letter code][genome – A or C][gene – ARF10].[locus].[allele – in this case E617 or 1012-98].

Locus/Allele	*At.ARF10*	*BraA.ARF10*	*BnaA.ARF10.a.E617*	*BnaA.ARF10.b.E617*	*BnaA.ARF10.b.1012-98*	*BnaAC.ARF10.a.1012-98*	*BolC.ARF10*	*BnaC.ARF10.a.E617*	*BnaC.ARF10.b.E617*	*BnaC.ARF10.a.1012-98*	*BnaC.ARF10.b.1012-98*	*BnaC.ARF10.c.1012-98*	*BnaC.ARF10.d.1012-98*
At.ARF10		85/88	85/88	85/88	85/88	85/88	85/88	85/88	85/88	85/88	85/88	85/88	85/88
BraA.ARF10	91		99/99	99/99	99/99	98/98	97/97	97/97	97/97	97/97	97/97	97/97	97/97
BnaA.ARF10.a.E617	95	99		100/100	100/100	98/99	97/97	97/97	97/97	97/97	97/97	97/97	97/97
BnaA.ARF10.b.E617	95	99	100		100/100	98/99	97/97	97/98	97/98	97/98	97/97	97/98	97/97
BnaA.ARF10.b.1012-98	95	99	100	100		98/99	97/97	97/98	97/98	97/98	97/97	97/98	97/97
BnaAC.ARF10.a.1012-98	95	98	99	100	100		98/98	99/99	99/98	99/99	99/98	99/98	99/98
BolC.ARF10	91	98	98	98	98	98		100/100	99/99	99/100	99/99	99/99	99/99
BnaC.ARF10.a.E617	95	98	99	99	99	100	99		100/100	100/100	100/100	100/100	100/100
BnaC.ARF10.b.E617	95	98	99	99	99	100	99	100		100/100	100/100	100/100	100/100
BnaC.ARF10.a.1012-98	95	98	99	99	99	100	99	100	100		100/100	100/100	100/100
BnaC.ARF10.b.1012-98	95	98	99	99	99	100	99	100	100	100		100/100	100/100
BnaC.ARF10.c.1012-98	95	98	99	99	99	100	99	100	100	100	100		100/100
BnaC.ARF10.d.1012-98	90	98	99	99	99	99	99	100	100	100	100	100	
